# Remoção de Cabos Eletrodos Transvenosos de Estimulação Cardíaca Artificial

**DOI:** 10.36660/abc.20210204

**Published:** 2021-05-06

**Affiliations:** 1 Hospital da Beneficência Portuguesa de São Paulo Centro Avançado de Ritmologia e Eletrofisiologia São PauloSP Brasil Centro Avançado de Ritmologia e Eletrofisiologia (CARE) - Hospital da Beneficência Portuguesa de São Paulo, São Paulo, SP – Brasil

**Keywords:** Cabo-eletrodo de Estimulação Cardíaca Artificial, Remoção percutânea, Infecção de sistemas de Estimulação Cardíaca, Ferramentas de extração, Dispositivos de Ressincronização Cardiaca

Os cabos eletrodos são a parte mais frágil dos sistemas de estimulação cardíaca artificial, sendo responsáveis pela maioria das complicações. A remoção dos cabos eletrodos transvenosos sempre foi um desafio, sendo que durante muito tempo a remoção percutânea era reservada aos eletrodos mais recentemente implantados e a toracotomia era a melhor opção para os casos mais antigos

A partir da década de 90, foram desenvolvidas várias ferramentas para remoção de eletrodos, tais como: guias especiais de aço com travas (locking stylet), bainhas de contra-tração sem ou com mecanismos de liberação mecânicos ou energizados a Laser e bainhas longas deflectíveis com guias Snare para extração via femoral. Esses instrumentos viabilizaram a extração percutânea de eletrodos mais antigos com índices altos de sucesso e baixos de complicações, como demonstrado no estudo europeu Electra^1^ que envolveu 3.510 pacientes apresentando 96,7% de sucesso clinico e 1,7% de complicações maiores. Com o aumento das indicações e a maior complexidade dos sistemas de estimulação cardíaca artificial (ECA)^2^ que passaram a necessitar por vezes de até 4 eletrodos, além da maior sobrevida dos seus portadores o que implica em várias trocas de gerador, a necessidade de remoção de eletrodos, por vezes mandatória, aumentou consideravelmente.

Não obstante esses enormes avanços, a remoção percutânea persiste sendo um procedimento complexo que envolve riscos. Portanto para realização desses procedimentos, alguns aspectos e devem ser considerados:

1. Indicação: Em algumas situações de ECA, como nas infecções, a indicação de remoção dos eletrodos é mandatória, já em outras a remoção completa pode ser discutível. Entretanto, em todos os casos, deve-se avaliar criteriosamente os riscos e benefício tanto da indicação como na escolha da modalidade de remoção.
2. Expertise do grupo: A experiência dos operadores com a utilização das diversas ferramentas de remoção percutânea de eletrodos, é fundamental para se obter bons resultados. Algumas diretrizes internacionais preconizam a realização de 40 extrações de eletrodos em no mínimo 30 intervenções, para qualificar o médico a realizar esses procedimentos.^3^^,^^4^ O Estudo Electra^1^ envolveu 73 centros em 19 países europeus e mostrou que centros de maior volume, definidos como os que realizam mais de 30 procedimentos de remoção por ano, apresentam significativos melhores resultados tanto em sucesso como complicações.
3. Disponibilidade de materiais: A remoção percutânea de cabos eletrodos com mais de um ano de implante, pressupõe a utilização de pelo menos um sistema de extração, quer por via subclávia-cava superior (bainhas de contra-tração com mecanismos de liberação com rotação mecânica ou energizada a laser), ou por via femoral-cava inferior (Bainhas longas deflectíveis com guias Snare).^5^ O ideal é que o grupo operador tenha experiência com as duas vias de acesso, já que a extração por via femoral pode ser complementar aquela por via subclávia, e em alguns casos de eletrodos abandonados é a única opção de extração percutânea. É importante também ter disponível cateteres balão de oclusão venosa (Bridge ballon) para casos de lesões graves no sistema venoso.
4. Condições do centro: O procedimento de extração percutânea deve ser realizado sob anestesia geral, com retaguarda cirúrgica (cardiovascular) e vaga na UTI, onde o paciente deve permanecer pelo menos no POi. O centro deverá ainda ter disponibilidade de ecocardiografia, transtorácica ou intracardíaca.

O artigo “Remoção Percutânea de Eletrodos de Estimulação Cardíaca Artificial em um Único Centro Sul-Americano”^6^ é uma das poucas publicações na literatura nacional mostrando a experiência inicial do serviço de um hospital público no Brasil, com remoção de 128 eletrodos em 61 pacientes, apresentando bons resultados (91% de sucesso clínico e 78,7% de sucesso total) e índices baixos de complicações (6,6% de complicações maiores sendo 3,3% de óbitos). Recentemente Costa et al.,^7^ publicou nessa revista um robusto registro prospectivo de remoção de eletrodos em um dos maiores centros cardiológicos do Brasil, envolvendo 634 eletrodos em 365 pacientes, utilizando todas as modalidades e ferramentas de extração e mostrando resultados bem melhores (96,7% de sucesso clínico e 90,1% de sucesso total) porém com maior mortalidade (8,2% de óbitos hospitalares dos quais somente 1,5% diretamente relacionados ao procedimento de extração). Acredito que esses dois artigos nacionais possam estimular a remoção percutânea de cabos eletrodos no Brasil, procedimento importante na estimulação cardíaca artificial e ainda subutilizado em nosso país.

A remoção de cabos eletrodos transvenosos é de longe o mais complexo e o que envolve maior risco dentre os procedimentos de estimulação cardíaca artificial. Utilizando-se adequadamente as ferramentas que dispomos atualmente, a remoção percutânea é a melhor opção na grande maioria dos casos, sendo um procedimento seguro e muito eficaz. Entretanto, a experiência do grupo operador é fundamental para obtenção de bons resultados e nesse sentido, nos primeiros procedimentos utilizando esses sistemas de extração, é muito importante contar com apoio de médicos habilitados para treinamento em regime de proctoria, até o grupo operador adquirir experiência.

## References

[1] 1. Bongiorni MG, Kennergren C, Butter C,Deharo JC, Kurtanski A,Rinaldi CA, et al. et al. The European Lead Extraction ConTRolled (ELECTRa) study: a European Heart Rhythm Association (EHRA) Registry of Transvenous Lead Extraction Outcomes. Eur Heart J. 2017;38:2995-3005. 10.1093/eurheartj/ehx080 28369414

[2] 2. Greenspon AJ, Patel JD, Lau E, Ochoa JA, Frisch DR, Ho RT, Pauri BB, Kurtz SM. Trends in permanent pacemaker implantation in the United States from 1993 to 2009: increasing complexity of patients and procedures. J Am Coll Cardiol. 2012;60:1540-1545. 10.1016/j.jacc.2012.07.017 22999727

[3] 3. Wilkoff BL, Love CJ, Byrd CL, Bongiorniet MG, Carrilo RG, Crossley GH, et al. The European Lead Straction: Heart Rhythm Society expert consensus of facilities, training, indication and patient management: This document was endorsed by the American Heart Association (AHA). Heart Rhythm. 2009;6:1085-104. 10.1016/j.hrthm.2009.05.020 19560098

[4] 4. Deharo JC, Bongiorni MG, Rozkovec A, Bracke F, Defayeet I. Pathway for traning and accreditation for transvenous lead extraction: a European Heart Rhythm Association position paper. Europace. 2012;14:124-34. 10.1093/europace/eur338 22167387

[5] 5. Bongiorni MG, Blomstrom-Lundquist C, Kennegreen C, Fernandez-Lozano P. Current practice in transvenous lead extraction: a European Heart Rhythm Association EP Network Survey. Europace. 2012;14(6):783-6. 10.1093/europace/eus166 22622992

[6] 6. Di Nubila BCLS, Lacerda GC, Rey HCV, Barbosa RM. Percutaneous Removal of Cardiac Leads in a Single Center in South America. Arq Bras Cardiol. 2021; 116(5):908-916. 10.36660/abc.20190726 PMC8121483 34008813

[7] 7. Costa R, Silva KR, Crevelari ES, Nascimento WTJ, Nagumo MM, Martinelli Filho M, Jatene FB. Efetividade e Segurança da Remoção de Cabos-Eletrodos Transvenosos de Marca-Passos e Desfibriladores Implantáveis no Cenário da Prática Clínica Real. Arq Bras Cardiol. 2020;115(6):1114-24. 10.36660/abc.20200476 PMC8133723 33470310

