# Supplementary material for: Remoção de Cabos Eletrodos Transvenosos de Estimulação Cardíaca Artificial
Source: Arq Bras Cardiol. 2021 May 6;116(5):917–8. [Article in Portuguese] doi: 10.36660/abc.20210204 (PMC8121474; doi:10.36660/abc.20210204)
Supplement: Translation

# Translation: Removal of Transvenous Pacing Leads in Artificial Cardiac Stimulation Systems

**Affiliations:** 1 São PauloSP Brazil Centro Avançado de Ritmologia e Eletrofisiologia (CARE) - Hospital da Beneficência Portuguesa de São Paulo, São Paulo, SP – Brazil

**Keywords:** Pacing Leads in Artificial Cardiac Stimulation, Percutaneous Removal, Infection of Cardiac Stimulation Systems, Extraction Tools, Cardiac Resynchronization Devices

The pacing leads are the most fragile part of the artificial cardiac stimulation systems, being responsible for the majority of complications. The removal of transvenous pacing leads has always been a challenge, and for a long time the percutaneous removal was reserved for the most recently implanted leads and thoracotomy was the best option for the older cases.

Since the 1990s, several tools for removing leads have been developed, such as: special stainless steel guide wires with locks (locking stylet), counter-traction sheaths without or with mechanical release mechanisms or laser-powered ones and long deflectable sheaths with snare guides for femoral extraction. These instruments allowed the percutaneous extraction of older leads with high success and low complication rates, as demonstrated in the European Electra Study,^1^ which involved 3,510 patients with 96.7% of clinical success and 1.7% of major complications. With the increase in indications and greater complexity of artificial cardiac stimulation (ACS)^2^ systems, which sometimes required up to 4 leads, in addition to the longer survival of the patients, which implies in several generator exchanges, the need for lead removal, sometimes mandatory, has considerably increased.

Despite these enormous advances, percutaneous removal remains a complex procedure that involves risks. Therefore, in order to carry out these procedures, some aspects must be considered:

1. Indication: In some situations of ACS, as in the presence of infections, the indication of lead removal is mandatory, while in others the complete removal may be debatable. However, in all cases, the risks and benefits of both the indication and the choice of removal method should be carefully evaluated.
2. Group expertise: The operators’ experience regarding the use of the several percutaneous lead removal tools is essential to achieve good results. Some international guidelines recommend the performance of 40 lead extractions in at least 30 interventions, to consider the physician qualified to perform these procedures.^3^^,^^4^ The Electra Study^1^ involved 73 centers in 19 European countries and showed that higher volume centers, defined as the ones that perform more than 30 removal procedures per year, have significantly better results regarding both success and complication rates.
3. Availability of materials: The percutaneous removal of lead cables with more than one year of implantation presupposes the use of at least one extraction system, either through the upper subclavian-cava route (counter-traction sheaths with release mechanisms with mechanical rotation or laser-powered ones), or via the femoral vena cava (long deflectable sheaths with snare guides).^5^ Ideally, the operator group should have experience with both access routes, since femoral extraction can be complementary that by the subclavian route, and in some cases of abandoned leads, it is the only option for percutaneous extraction. It is also important to have venous occlusion-balloon catheters (Bridge balloon) available, for cases of severe lesions in the venous system.
4. Operating Center conditions: The percutaneous extraction procedure must be performed under general anesthesia, with surgical (cardiovascular) support and a place in the ICU, where the patient must remain at least during the immediate postoperative (IPO) period. The center should also have transthoracic or intracardiac echocardiography available.

The article “Percutaneous Removal of Cardiac Leads in a Single Center in South America”^6^ is one of the few publications in the national literature showing the initial experience of a public hospital service in Brazil, with the removal of 128 leads in 61 patients, showing good results (91% of clinical success and 78.7% of total success) and low complication rates (6.6% of major complications, 3.3% of deaths). Recently Costa et al.^7^ published in this journal a robust prospective registry of lead removal in one of the largest cardiological centers in Brazil, involving 634 leads in 365 patients, using all the modalities and extraction tools and showing much better results (96.7% of clinical success and 90.1% of total success) but with higher mortality (8.2% of in-hospital deaths, of which only 1.5% are directly related to the extraction procedure). I believe that these two national articles can stimulate the percutaneous removal of lead cables in Brazil, an important procedure in artificial cardiac pacing and still underutilized in our country.

The removal of transvenous lead cables is by far the most complex and the one involving the greatest risk among artificial cardiac pacing procedures. Using the tools that are currently available, percutaneous removal is the best option in the vast majority of cases, being a safe and very effective procedure. However, the experience of the operator group is fundamental to obtain good results and, in this sense, regarding the first procedures using these extraction systems, it is very important to have the support of physicians qualified for training under the proctoring regime, until the operator group has gained experience.
